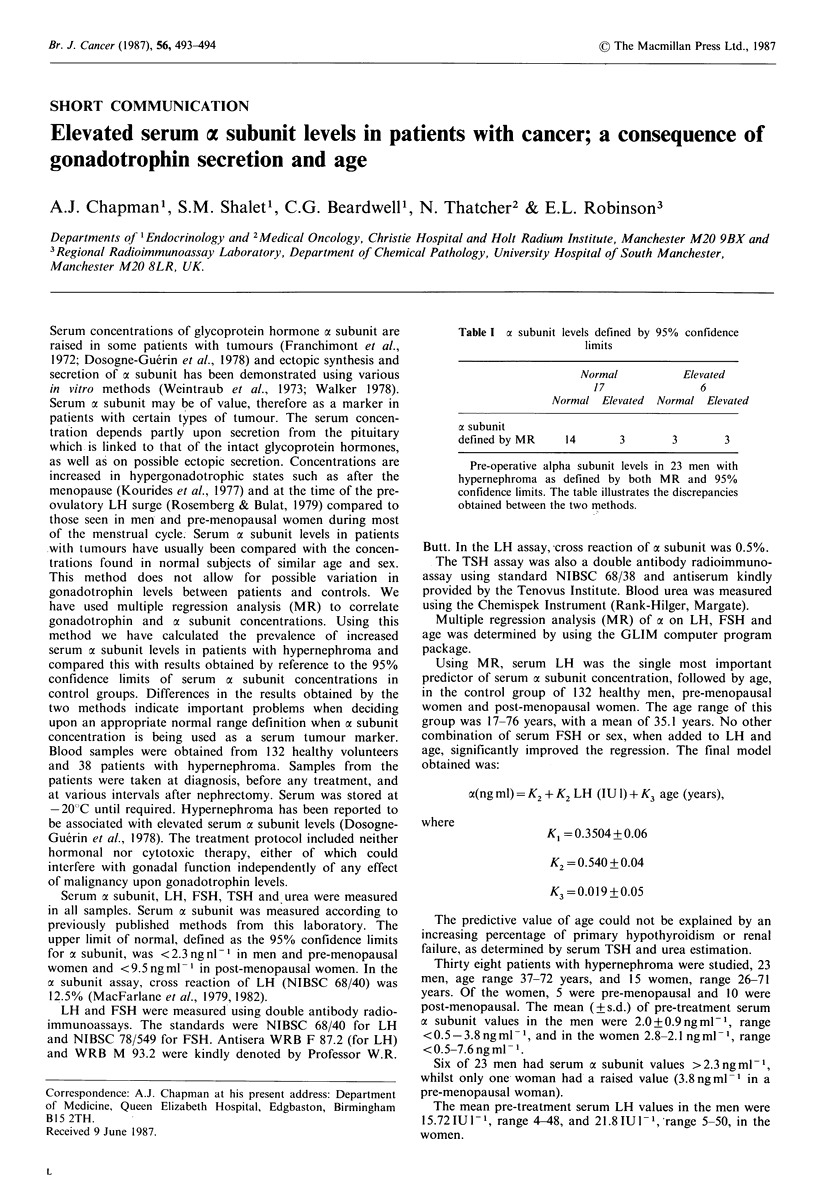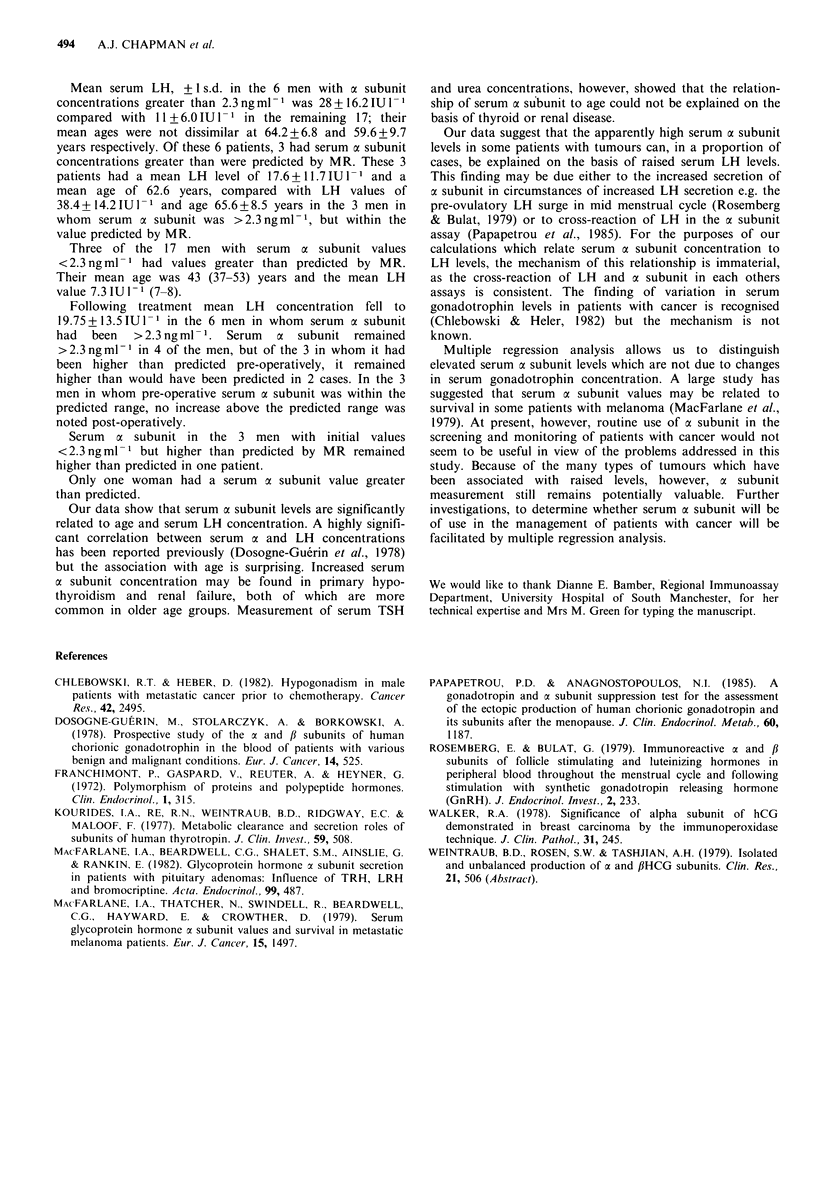# Elevated serum alpha subunit levels in patients with cancer; a consequence of gonadotrophin secretion and age.

**DOI:** 10.1038/bjc.1987.231

**Published:** 1987-10

**Authors:** A. J. Chapman, S. M. Shalet, C. G. Beardwell, N. Thatcher, E. L. Robinson

**Affiliations:** Department of Endocrinology, Christie Hospital, Manchester, UK.


*Br. J. Cancer* (1987), **56**, 493–494 © The Macmillan Press Ltd., 1987

SHORT COMMUNICATION

### Elevated serum α subunit levels in patients with cancer; a consequence of gonadotrophin secretion and age

A.J. Chapman[1], S.M. Shalet[1], C.G. Beardwell[1], N. Thatcher[2] & E.L. Robinson[3]

*Departments of [1]Endocrinology and [2]Medical Oncology, Christie Hospital and Holt Radium Institute, Manchester M20 9BX and [3]Regional Radioimmunoassay Laboratory, Department of Chemical Pathology, University Hospital of South Manchester, Manchester M20 8LR, UK.*

Serum concentrations of glycoprotein hormone α subunit are raised in some patients with tumours (Franchimont *et al.*, 1972; Dosogne-Guérin *et al.*, 1978) and ectopic synthesis and secretion of α subunit has been demonstrated using various *in vitro* methods (Weintraub *et al.*, 1973; Walker 1978). Serum α subunit may be of value, therefore as a marker in patients with certain types of tumour. The serum concentration depends partly upon secretion from the pituitary which is linked to that of the intact glycoprotein hormones, as well as on possible ectopic secretion. Concentrations are increased in hypergonadotrophic states such as after the menopause (Kourides *et al.*, 1977) and at the time of the pre-ovulatory LH surge (Rosemberg & Bulat, 1979) compared to those seen in men and pre-menopausal women during most of the menstrual cycle. Serum α subunit levels in patients with tumours have usually been compared with the concentrations found in normal subjects of similar age and sex. This method does not allow for possible variation in gonadotrophin levels between patients and controls. We have used multiple regression analysis (MR) to correlate gonadotrophin and α subunit concentrations. Using this method we have calculated the prevalence of increased serum α subunit levels in patients with hypernephroma and compared this with results obtained by reference to the 95% confidence limits of serum α subunit concentrations in control groups. Differences in the results obtained by the two methods indicate important problems when deciding upon an appropriate normal range definition when α subunit concentration is being used as a serum tumour marker. Blood samples were obtained from 132 healthy volunteers and 38 patients with hypernephroma. Samples from the patients were taken at diagnosis, before any treatment, and at various intervals after nephrectomy. Serum was stored at −20°C until required. Hypernephroma has been reported to be associated with elevated serum α subunit levels (Dosogne-Guérin *et al.*, 1978). The treatment protocol included neither hormonal nor cytotoxic therapy, either of which could interfere with gonadal function independently of any effect of malignancy upon gonadotrophin levels.

Serum α subunit, LH, FSH, TSH and urea were measured in all samples. Serum α subunit was measured according to previously published methods from this laboratory. The upper limit of normal, defined as the 95% confidence limits for α subunit, was $<2.3\,\mathrm{ng\,nl^{-1}}$ in men and pre-menopausal women and $<9.5\,\mathrm{ng\,ml^{-1}}$ in post-menopausal women. In the α subunit assay, cross reaction of LH (NIBSC 68/40) was 12.5% (MacFarlane *et al.*, 1979, 1982).

LH and FSH were measured using double antibody radioimmunoassays. The standards were NIBSC 68/40 for LH and NIBSC 78/549 for FSH. Antisera WRB F 87.2 (for LH) and WRB M 93.2 were kindly denoted by Professor W.R. Butt. In the LH assay, cross reaction of α subunit was 0.5%.

Correspondence: A.J. Chapman at his present address: Department of Medicine, Queen Elizabeth Hospital, Edgbaston, Birmingham B15 2TH.
Received 9 June 1987.

**Table I** α subunit levels defined by 95% confidence limits

| | *Normal* 17 | | *Elevated* 6 | |
|---|---|---|---|---|
| | *Normal* | *Elevated* | *Normal* | *Elevated* |
| α subunit defined by MR | 14 | 3 | 3 | 3 |

Pre-operative alpha subunit levels in 23 men with hypernephroma as defined by both MR and 95% confidence limits. The table illustrates the discrepancies obtained between the two methods.

The TSH assay was also a double antibody radioimmunoassay using standard NIBSC 68/38 and antiserum kindly provided by the Tenovus Institute. Blood urea was measured using the Chemispek Instrument (Rank-Hilger, Margate).

Multiple regression analysis (MR) of α on LH, FSH and age was determined by using the GLIM computer program package.

Using MR, serum LH was the single most important predictor of serum α subunit concentration, followed by age, in the control group of 132 healthy men, pre-menopausal women and post-menopausal women. The age range of this group was 17–76 years, with a mean of 35.1 years. No other combination of serum FSH or sex, when added to LH and age, significantly improved the regression. The final model obtained was:

$$\alpha(\mathrm{ng\,ml}) = K_2 + K_2\,\mathrm{LH}\ (\mathrm{IU\,l}) + K_3\ \mathrm{age\ (years)},$$

where

$$K_1 = 0.3504 \pm 0.06$$

$$K_2 = 0.540 \pm 0.04$$

$$K_3 = 0.019 \pm 0.05$$

The predictive value of age could not be explained by an increasing percentage of primary hypothyroidism or renal failure, as determined by serum TSH and urea estimation.

Thirty eight patients with hypernephroma were studied, 23 men, age range 37–72 years, and 15 women, range 26–71 years. Of the women, 5 were pre-menopausal and 10 were post-menopausal. The mean (±s.d.) of pre-treatment serum α subunit values in the men were $2.0 \pm 0.9\,\mathrm{ng\,ml^{-1}}$, range $<0.5-3.8\,\mathrm{ng\,ml^{-1}}$, and in the women $2.8–2.1\,\mathrm{ng\,ml^{-1}}$, range $<0.5–7.6\,\mathrm{ng\,ml^{-1}}$.

Six of 23 men had serum α subunit values $>2.3\,\mathrm{ng\,ml^{-1}}$, whilst only one woman had a raised value ($3.8\,\mathrm{ng\,ml^{-1}}$ in a pre-menopausal woman).

The mean pre-treatment serum LH values in the men were $15.72\,\mathrm{IU\,l^{-1}}$, range 4–48, and $21.8\,\mathrm{IU\,l^{-1}}$, range 5–50, in the women.

Mean serum LH, $\pm 1$ s.d. in the 6 men with $\alpha$ subunit concentrations greater than $2.3\,ng\,ml^{-1}$ was $28 \pm 16.2\,IU\,l^{-1}$ compared with $11 \pm 6.0\,IU\,l^{-1}$ in the remaining 17; their mean ages were not dissimilar at $64.2 \pm 6.8$ and $59.6 \pm 9.7$ years respectively. Of these 6 patients, 3 had serum $\alpha$ subunit concentrations greater than were predicted by MR. These 3 patients had a mean LH level of $17.6 \pm 11.7\,IU\,l^{-1}$ and a mean age of 62.6 years, compared with LH values of $38.4 \pm 14.2\,IU\,l^{-1}$ and age $65.6 \pm 8.5$ years in the 3 men in whom serum $\alpha$ subunit was $> 2.3\,ng\,ml^{-1}$, but within the value predicted by MR.

Three of the 17 men with serum $\alpha$ subunit values $< 2.3\,ng\,ml^{-1}$ had values greater than predicted by MR. Their mean age was 43 (37–53) years and the mean LH value $7.3\,IU\,l^{-1}$ (7–8).

Following treatment mean LH concentration fell to $19.75 \pm 13.5\,IU\,l^{-1}$ in the 6 men in whom serum $\alpha$ subunit had been $> 2.3\,ng\,ml^{-1}$. Serum $\alpha$ subunit remained $> 2.3\,ng\,ml^{-1}$ in 4 of the men, but of the 3 in whom it had been higher than predicted pre-operatively, it remained higher than would have been predicted in 2 cases. In the 3 men in whom pre-operative serum $\alpha$ subunit was within the predicted range, no increase above the predicted range was noted post-operatively.

Serum $\alpha$ subunit in the 3 men with initial values $< 2.3\,ng\,ml^{-1}$ but higher than predicted by MR remained higher than predicted in one patient.

Only one woman had a serum $\alpha$ subunit value greater than predicted.

Our data show that serum $\alpha$ subunit levels are significantly related to age and serum LH concentration. A highly significant correlation between serum $\alpha$ and LH concentrations has been reported previously (Dosogne-Guérin *et al.*, 1978) but the association with age is surprising. Increased serum $\alpha$ subunit concentration may be found in primary hypothyroidism and renal failure, both of which are more common in older age groups. Measurement of serum TSH and urea concentrations, however, showed that the relationship of serum $\alpha$ subunit to age could not be explained on the basis of thyroid or renal disease.

Our data suggest that the apparently high serum $\alpha$ subunit levels in some patients with tumours can, in a proportion of cases, be explained on the basis of raised serum LH levels. This finding may be due either to the increased secretion of $\alpha$ subunit in circumstances of increased LH secretion e.g. the pre-ovulatory LH surge in mid menstrual cycle (Rosemberg & Bulat, 1979) or to cross-reaction of LH in the $\alpha$ subunit assay (Papapetrou *et al.*, 1985). For the purposes of our calculations which relate serum $\alpha$ subunit concentration to LH levels, the mechanism of this relationship is immaterial, as the cross-reaction of LH and $\alpha$ subunit in each others assays is consistent. The finding of variation in serum gonadotrophin levels in patients with cancer is recognised (Chlebowski & Heler, 1982) but the mechanism is not known.

Multiple regression analysis allows us to distinguish elevated serum $\alpha$ subunit levels which are not due to changes in serum gonadotrophin concentration. A large study has suggested that serum $\alpha$ subunit values may be related to survival in some patients with melanoma (MacFarlane *et al.*, 1979). At present, however, routine use of $\alpha$ subunit in the screening and monitoring of patients with cancer would not seem to be useful in view of the problems addressed in this study. Because of the many types of tumours which have been associated with raised levels, however, $\alpha$ subunit measurement still remains potentially valuable. Further investigations, to determine whether serum $\alpha$ subunit will be of use in the management of patients with cancer will be facilitated by multiple regression analysis.

We would like to thank Dianne E. Bamber, Regional Immunoassay Department, University Hospital of South Manchester, for her technical expertise and Mrs M. Green for typing the manuscript.

### References

CHLEBOWSKI, R.T. & HEBER, D. (1982). Hypogonadism in male patients with metastatic cancer prior to chemotherapy. *Cancer Res.*, **42**, 2495.

DOSOGNE-GUÉRIN, M., STOLARCZYK, A. & BORKOWSKI, A. (1978). Prospective study of the $\alpha$ and $\beta$ subunits of human chorionic gonadotrophin in the blood of patients with various benign and malignant conditions. *Eur. J. Cancer*, **14**, 525.

FRANCHIMONT, P., GASPARD, V., REUTER, A. & HEYNER, G. (1972). Polymorphism of proteins and polypeptide hormones. *Clin. Endocrinol.*, **1**, 315.

KOURIDES, I.A., RE, R.N., WEINTRAUB, B.D., RIDGWAY, E.C. & MALOOF, F. (1977). Metabolic clearance and secretion roles of subunits of human thyrotropin. *J. Clin. Invest.*, **59**, 508.

MACFARLANE, I.A., BEARDWELL, C.G., SHALET, S.M., AINSLIE, G. & RANKIN, E. (1982). Glycoprotein hormone $\alpha$ subunit secretion in patients with pituitary adenomas: Influence of TRH, LRH and bromocriptine. *Acta. Endocrinol.*, **99**, 487.

MACFARLANE, I.A., THATCHER, N., SWINDELL, R., BEARDWELL, C.G., HAYWARD, E. & CROWTHER, D. (1979). Serum glycoprotein hormone $\alpha$ subunit values and survival in metastatic melanoma patients. *Eur. J. Cancer*, **15**, 1497.

PAPAPETROU, P.D. & ANAGNOSTOPOULOS, N.I. (1985). A gonadotropin and $\alpha$ subunit suppression test for the assessment of the ectopic production of human chorionic gonadotropin and its subunits after the menopause. *J. Clin. Endocrinol. Metab.*, **60**, 1187.

ROSEMBERG, E. & BULAT, G. (1979). Immunoreactive $\alpha$ and $\beta$ subunits of follicle stimulating and luteinizing hormones in peripheral blood throughout the menstrual cycle and following stimulation with synthetic gonadotropin releasing hormone (GnRH). *J. Endocrinol. Invest.*, **2**, 233.

WALKER, R.A. (1978). Significance of alpha subunit of hCG demonstrated in breast carcinoma by the immunoperoxidase technique. *J. Clin. Pathol.*, **31**, 245.

WEINTRAUB, B.D., ROSEN, S.W. & TASHJIAN, A.H. (1979). Isolated and unbalanced production of $\alpha$ and $\beta$HCG subunits. *Clin. Res.*, **21**, 506 (*Abstract*).